# Salvage Radiotherapy in Isolated Locoregional Recurrence of Pancreatic Adenocarcinoma Post-Radical Surgical Resection: Prudent or Pointless? A Retrospective Comparative Analysis

**DOI:** 10.3390/curroncol33060337

**Published:** 2026-06-06

**Authors:** Colin Faulkner, Ayah Erjan, Sara Mheid, Michael Yan, Chaya Ganor Shwaartz, Erica Tsang, Sangeetha Kalimuthu, Teodor Stanescu, Ali Hosni, Aruz Mesci

**Affiliations:** 1 Radiation Medicine Program, Princess Margaret Cancer Centre, Toronto, ON M5G 2C4, Canada; 2 Department of Radiation Oncology, King Hussein Cancer Center, Amman 11941, Jordan; 3 Department of Surgery, University of Toronto, Toronto, ON M5T 1P5, Canada; 4 Medical Oncology, Princess Margaret Cancer Centre, University of Toronto, Toronto, ON M5G 2M9, Canada; 5 Laboratory Medicine Program, Princess Margaret Cancer Centre, Toronto, ON M5G 2C4, Canada

**Keywords:** pancreatic cancer, locoregional recurrence, radiotherapy

## Abstract

Isolated Locoregionally Recurrent Pancreatic Adenocarcinoma (ILRPA) accounts for 25–30% of recurrences after radical resection, yet the role of salvage radiation (RT) in this setting remains controversial due to limited data. We aimed to evaluate the impact of salvage RT on survival outcomes compared with non-salvage RT in patients with ILRPA. We retrospectively analyzed data of patients with ILRPA post-radical resection treated at our centre between 2012 and 2021. Patients were categorized into two cohorts based on whether salvage RT was administered post-recurrence. Patients who received a minimum of 30 Gy in 10 fractions (BED_10_ ≥ 39 Gy) were included for analysis. The clinical characteristics, treatments, and outcomes were analyzed. The chi-square or Fisher’s exact test for categorical variables and Student’s *t*-test or Wilcoxon rank sum test for continuous variables were utilized for comparisons. The Kaplan–Meier method and log-rank test were performed to compare overall survival (OS) and progression-free survival (PFS) between the two groups. OS and PFS were calculated from the time of locoregional recurrence until event or loss of follow-up. Thirty-two patients were identified, with sixteen patients in each group. The patients and tumour characteristics were balanced between the two cohorts, except for chemotherapy, where the salvage RT group tended to receive palliative chemotherapy more than the non-salvage group (*p* = 0.007). The median radiotherapy dose received for the salvage RT cohort was 50.4 Gy (BED_10_ = 59.5 Gy). Chemotherapy was restarted in 75% either before, after, or during radiation, and only in 31.25% of patients in the non-salvage group (*p* = 0.013). Patients who received salvage RT had statistically significantly better median OS and PFS than those who did not (25.2 vs. 8.4 months, *p* = 0.0006, HR 0.25, 95% CI (0.11–0.59)), (15.6 vs. 7.2 months, *p* = 0.0006, HR 0.26, 95% CI (0.11–0.58), respectively). Ten patients (62.5%) developed distant metastases (DM) at least 3 months post-recurrence in the salvage RT cohort compared to five patients (31.25%) in the non-salvage RT cohort (*p* = 0.08). In the salvage RT group, 10/16 patients (62.5%) maintained locoregional disease control post-RT. Salvage RT was associated with better OS and PFS in patients with ILRPA, highlighting its potential as an essential treatment modality. While salvage RT appears beneficial, confounding factors like chemotherapy disparities between groups necessitate further investigation in prospective cohorts.

## Simple Summary

Isolated Locoregionally Recurrent Pancreatic Adenocarcinoma presents a challenging clinical scenario, and the role of radiotherapy is not well established. In this study, we reviewed outcomes of patients with isolated locoregionally recurrent pancreatic cancer treated with radiotherapy with an informal comparison to a group not treated with RT. Our results suggest that radiotherapy is associated with greater overall and progression-free survival. Multi-disciplinary assessment of such patients is key, and for well-selected patients, radiotherapy should be part of the treatment considerations where appropriate.

## 1. Introduction

Pancreatic ductal adenocarcinoma (PDAC) is a challenging disease with high morbidity and mortality [1]. Surgery is fundamental to the curative management of PDAC. Neoadjuvant and adjuvant therapies reduce the risk of distant/locoregional recurrence and improve the resectability of the primary disease [2,3]. Nevertheless, patients remain at high risk of disease recurrence, typically in the form of distant metastases. However, isolated locoregional recurrence accounts for 25–30% [4,5] of recurrences. In these cases, patients typically still receive systemic therapy given that their risk of distant recurrence is high. A systematic review and meta-analysis investigated the feasibility of re-resection of Isolated Locoregional Recurrent Pancreatic Adenocarcinoma (ILRPA, i.e., without distant metastasis); among 176 patients who underwent re-resection compared to 255 who received non-surgical therapies, significantly better OS and median survival rates were seen following re-resection compared to other treatment modalities [6], highlighting the importance of local therapies in the recurrent setting. However, repeat surgery may not be technically or clinically possible in such cases, when salvage radiation therapy (RT) offers a non-invasive alternative to improve locoregional control.

The question of whether salvage RT for ILRPA would be helpful (often post-chemotherapy) is frequently raised. A few studies suggest that high doses of radiotherapy, either in the form of stereotactic body radiation therapy (SBRT) or long-course concurrent chemoradiotherapy, may result in higher rates of local disease control [4,7,8,9]. However, distant relapse rates remain high. Our study aims to evaluate the outcomes of patients with ILRPA who received salvage RT and compare them to those with ILRPA who did not receive salvage RT at our institution. This study addresses the gap in comparative data by evaluating survival outcomes of salvage RT versus no RT in ILRPA, hypothesizing that RT may be associated with improved OS and PFS through enhanced locoregional control.

The abstract of this work was previously presented at the annual scientific meeting of ASTRO 2024 [10]. The present study provides expanded methodology, additional analyses, and complete results.

## 2. Materials and Methods

### 2.1. Study Design and Population

We retrospectively analyzed data of patients with ILRPA post-radical resection treated at our institution between 2012 and 2021. Patients aged 18 years or older with pathologically and/or radiologically confirmed ILRPA following curative surgical resection for primary pancreatic adenocarcinoma were eligible for the study. Patients who received palliative systemic treatments prior to or after salvage RT were included. We excluded patients with distant metastases at the time of locoregional recurrence (LRR), histology other than adenocarcinoma (e.g., neuroendocrine pathology, lymphoma, etc.), carcinoma of the ampulla of Vater, patients with no follow-up information, and those who underwent salvage surgery for ILRPA. Patients were categorized into two cohorts based on whether salvage RT was administered post-recurrence or not. Patients who received a minimum of 30 Gray in 10 fractions (Biological Effective Dose [BED_10_] ≥ 39 Gy) were included for analysis. The study was approved by the University Health Network’s (UHN) Research Ethics Board, and informed consent was waived, given the retrospective nature of the study. Patients’ charts were accessed from the Princess Margaret Cancer Centre (PMCC) Registry Database, which included data on patients diagnosed with pancreatic adenocarcinoma from 1 January 2012 to 1 January 2021, with follow-up extending to 1 February 2024.

### 2.2. Data Collection

We conducted comprehensive data extraction of the eligible patients’ medical charts. The following data were recorded: age at diagnosis, sex, pathologic characteristics at time of radical surgery prior to LLR (histologic grade, resection margin status, presence of lymphovascular invasion (LVI), location of the primary tumour, pathological TNM [pTNM] stage), systemic treatment approach for original disease, date of LRR, local/systemic treatment approaches on recurrence, and the patterns of LRR (in the remnant pancreas vs. surgical bed vs. near vascular structures). We also collected radiation dose/fractionation, Planning Target Volume (PTV) volume, dose received by the adjacent Organs-at-Risk (OAR), and the presence of Common Terminology Criteria for Adverse Events version 5 (CTCAEv5) grade III and higher toxicity in those who received RT on recurrence. Patients who met the study criteria were categorized into two cohorts: those who received salvage RT (a minimum of 30 Gy in 10 fractions (BED_10_ ≥ 39 Gy) was allowed) post-recurrence and those who did not.

### 2.3. Statistical Analysis

Descriptive analysis for the clinical characteristics, treatments, and outcomes was performed for both groups. Continuous variables were summarized using mean and standard deviation, or median and interquartile range, where appropriate. Categorical variables were presented as frequencies and percentages.

We compared patient characteristics between the two groups, those who received salvage RT and those who did not, using appropriate statistical tests. For continuous variables, we applied Student’s *t*-test for normally distributed data or the Wilcoxon rank-sum test for non-normally distributed data. For categorical variables, we used the chi-square test or Fisher’s exact test, depending on the expected frequencies. Outcomes, including mortality, were analyzed using similar statistical methods. Overall survival (OS) was calculated from the date of radiological evidence of locoregional recurrence until the date of death or last follow-up. Progression-free survival (PFS) was calculated from the date of radiological evidence of locoregional recurrence to locoregional disease progression and/or distant failure, death, or last follow-up. To compare OS and PFS between the two groups, we used the Kaplan–Meier method to generate survival curves, and the log-rank test was performed to assess differences between the groups. All statistical analyses were conducted using SAS v9.4. A two-sided *p*-value of <0.05 was considered statistically significant.

## 3. Results

### 3.1. Cohort Characteristics

**Figure 1:**
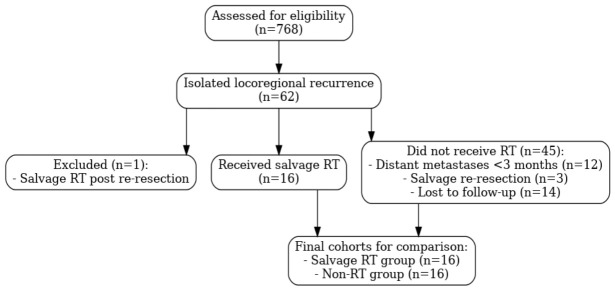
Prisma flowchart illustrating the selection and allocation of patients for the final analysis in the study.

Among 768 patients in the pancreatic cancer database, 62 developed isolated locoregional recurrence. Of these, 17 patients received salvage RT. However, one patient was excluded because RT was administered post-re-resection. Among the remaining 45 patients who did not receive RT, 12 developed distant metastases in less than 3 months, 3 underwent salvage re-resection, and 14 were lost to follow-up after the diagnosis of recurrence. Consequently, we included 16 patients in each cohort (salvage RT vs. no salvage RT), as illustrated in Figure 1. The equal number of patients in the two cohorts was purely coincidental.

### 3.2. Baseline Patient, Tumour, and Treatment Characteristics

Median age for the salvage RT cohort was 60.6 years (range, 44.4–78.0) compared to 67 years (range, 50.6–84.0) in the non-salvage group (*p* = 0.32). Fifty percent (n = 8) of the salvage RT cohort were males compared to 37.5% (n = 6) in the non-salvage cohort (*p* = 0.48). Head/ neck/uncinate process was the most common location for recurrence in both cohorts, comprising 93.75% (n = 15) and 87.5% (n = 14) in salvage and salvage RT groups, respectively. A negative resection margin (for radical surgery prior to LRR) was achieved in all patients in the salvage RT cohort, and all except one in the non-salvage cohort. LVI was present in 78.75% (n = 11) in the salvage RT group compared to 57.14% (n = 8) in the non-salvage RT group (*p* = 0.42). Most patients had elevated Ca19-9 levels prior to treatment (81% and 88% in salvage and non-salvage groups, respectively). Approximately 50% of the patients in the cohort underwent genetic testing. No BRCA mutations or actionable mutations were identified in those who underwent testing. Patient and tumour characteristics are summarized in Table 1.

Nearly all patients underwent initial Whipple surgery; one in the salvage RT group and three in the non-salvage RT group received distal pancreatectomy for primary pancreatic tail adenocarcinoma. All patients in the salvage RT group received chemotherapy (adjuvant and/or neoadjuvant), whereas 43.75% (n = 7) of the non-salvage RT group did not receive chemotherapy as a part of their initial treatment. Among the latter, two patients declined chemotherapy. FOLFIRINOX was the predominant chemotherapy regimen in the salvage RT group, while gemcitabine was most frequently administered in the non-salvage RT cohort. The mean duration of chemotherapy (in months) was shorter in the non-salvage RT cohort compared to the salvage RT cohort (1.69 mo vs. 4.7 mo; *p* < 0.001). Chemotherapy details are given in Table 2.

The median time from surgery to LRR for the entire cohort was 14.4 months (range, 4.8–84) with no significant difference between the two groups (*p* = 0.92). Patients in the non-salvage RT group tended to have somewhat larger recurrent disease size with a median recurrent focus of 2.10 cm in salvage RT cohort (range 1.2–3.9 cm); vs. 2.6 cm in the non-salvage cohort (range 1.0–5.0 cm; *p* = 0.033; Table 3), although the number of identified recurrent foci was similar between the two cohorts (median number of recurrent foci of 1.0. Patients in the salvage RT group received a median BED_10_ of 59.5 Gy (range: 39–61.7 Gy; Table 4). Most patients (12/16) received conventionally fractionated radiotherapy with a 1.8 Gy daily dose, concurrently with oral capecitabine. Only 3 patients received hypofractionated RT with a 3.0 Gy daily dose, and one received a low-dose SBRT regimen with 6.0 Gy per day, all to 30 Gy total. All except for one patient were treated with inverse planning techniques (IMRT/VMAT); one patient was treated with field-based techniques. For all patients, CT-simulation was obtained. Gross tumour volume (GTV) was contoured according to visible gross disease on imaging. In all patients but one, the celiac and superior-mesenteric artery axis was included in the clinical target volume (CTV), along with a 1 cm expansion on GTV delimited by intact barriers to spread. In one patient with recurrence in the distal pancreatectomy site, CTV consisted only of a 1 cm expansion without the vascular volumes. An internal target volume (ITV) was generated according to breathing phases (inhale and exhale), with a 5 mm expansion on the ITV to generate the planning target volume (PTV). Dosimetric parameters for the salvage RT cohort are summarized in Table 4. For treatment of recurrent disease, chemotherapy was administered in 75% (n = 12/16) of patients in the salvage RT group, compared to 31.25% (n = 5/16) in the non-salvage RT group (*p* = 0.013).

### 3.3. Outcomes

**Figure 2:**
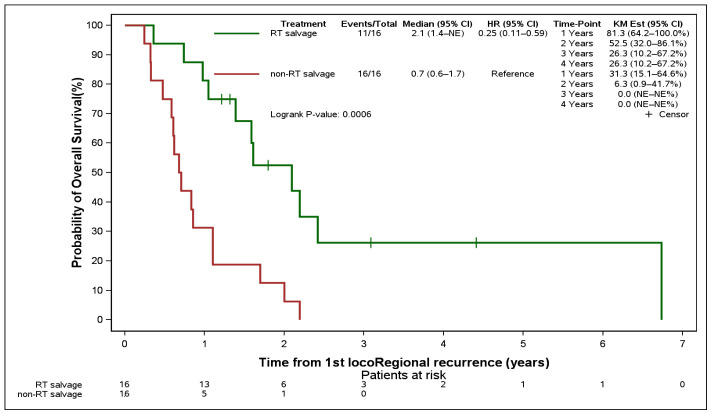
Kaplan–Meier curve of overall survival (OS) between two treatment groups (salvage RT and non-salvage RT).

**Figure 3:**
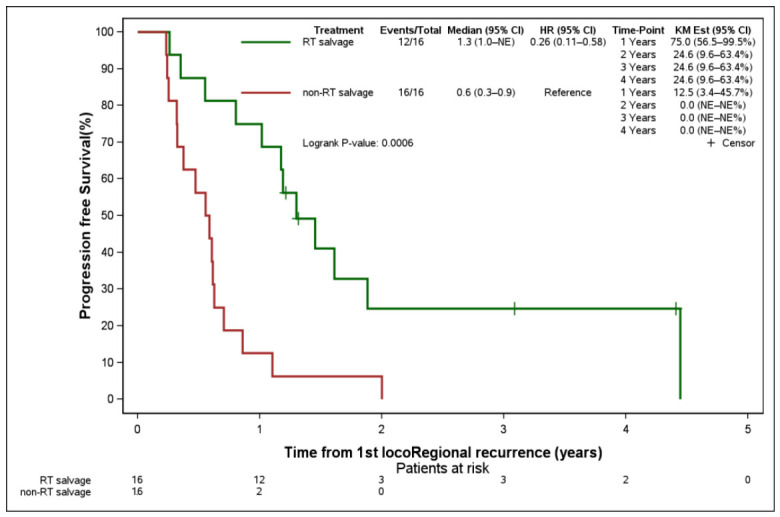
Kaplan–Meier curve of progression-free survival (PFS) between two treatment groups (salvage RT and non-salvage RT).

The median OS for the salvage RT group was significantly longer at 25.2 months (95% CI: 20.1–30.3) compared to 8.4 months (95% CI: 6.7–10.1) in the non-salvage RT group (*p* = 0.0006, HR 0.25, 95% CI [0.11–0.59]; Figure 2). The median progression-free survival (PFS) was also significantly longer in the salvage RT group at 15.6 months (95% CI: 12.4–18.8) compared to 7.2 months (95% CI: 5.6–8.8) in the non-salvage RT group (*p* = 0.0006, HR 0.26, 95% CI [0.11–0.58]; Figure 3). Regarding disease control, 10 patients (62.5%) of the salvage RT cohort developed distant metastases at least 3 months post-recurrence, compared to 5 patients (31.25%) in the non-salvage RT cohort (*p* = 0.08). Although the difference was not statistically significant, a trend towards increased metastasis in the salvage RT group was observed. Liver was the most common site of DM (occurred in 6 out of 10 patients), followed by the peritoneum and lung. In the salvage RT group, 10 out of 16 patients (62.5%) maintained locoregional disease control (LRC) after RT. However, in the non-salvage RT group, only one patient who received palliative chemotherapy had LRC. There was no statistically significant difference in OS between patients treated with conventional fractionation versus hypofractionation (*p* = 0.16), although the number of patients in the latter group is small. Biochemical response post radiotherapy was only evident in 4 out of the 13 salvage RT patients who had elevated Ca19-9 levels prior to radiotherapy. The remainder showed further rise. With respect to toxicity, no CTCAE grade III toxicities were recorded with the administration of salvage RT. Reported acute toxicities were limited to grade I (5/16 patients; nausea, anorexia, diarrhea) and II (3/16, nausea/vomiting and melena stools), all of which were managed with supportive care. One patient with melena stools had an interruption of radiotherapy for 4 weeks, but completed her course with no further complications. No significant late toxicities attributable to radiation were recorded. 5/16 patients reported significant worsening deterioration attributed to metastatic disease progression according to available imaging and clinical assessments.

## 4. Discussion

The results of this study suggest that the addition of salvage RT to palliative chemotherapy for ILRPA is associated with better OS and PFS. Strikingly, salvage RT appears to slow the course of the disease, with a much longer median PFS in the salvage RT group (15.6 months) than in the non-salvage RT group (7.2 months). Significant LRC was achieved within the cohort. Interestingly, there was a trend toward a higher incidence of distant metastases (62.5% versus 31.25%) in the salvage RT group, but this trend did not reach statistical significance (*p* = 0.08). This observation may be attributable to longer OS within this group, and for distant metastases to eventually declare themselves. Salvage RT, therefore, does not completely prevent or postpone the formation of distant metastases, even though it is effective at controlling locoregional recurrence. These patients are still vulnerable to the spread of systemic disease, and patients need to be considered for effective systemic therapies.

Our findings are consistent with previous research on the application of salvage therapy in comparable contexts [1,8,9,11,12,13]. For instance, 18 patients received RT (45 Gy in 25 fractions) in a study by Wilkowski et al. [9]; the patients’ median OS and PFS were 17.5 and 14.7 months, respectively. In a more recent study, 41 patients receiving RT (with a median dosage of 48.4 Gy, which included patients treated with IORT as well) were reported by Habermehl et al. [11]. The overall cohort’s median OS was 16.1 months. Our study’s median OS of 25.2 months in the salvage RT group appears superior to Habermehl’s overall cohort and comparable to their high-performing IORT subgroup. Additionally, Nakamura et al. retrospectively examined 30 patients who had RT (median dose 54 Gy) between 2000 and 2013 for ILRPA [1]. Some individuals had chemotherapy in addition to treatment. The 1-year OS rate of 69%, a 67% local control rate, and a 32% PFS rate were reported in the data. The PFS median was 6.9 months, and the OS rate was 15.9 months, respectively. In a 2022 prospective study, high-dose salvage RT was given to post-resection locoregional recurrence of PDAC. For 69 patients, OS and PFS was 91.5% and 78.5% at 12 months, and 72.5% and 40.3% at 24 months, respectively [4]. To provide further context, Table 5 summarizes key studies evaluating salvage RT approaches for ILRPA, highlighting variations in study design, sample size, radiation dose, and outcomes such as local control and survival metrics.

A key strength of our study is the inclusion of a comparative cohort of patients who did not receive salvage RT. Existing studies on this topic are single-arm retrospective analyses, making it difficult to quantify the true benefit of RT. Our indirect comparison provides stronger evidence for the significant improvement in both OS and PFS attributable to the addition of salvage RT and a higher proportion of patients receiving palliative chemotherapy. While our study primarily involved conventionally fractionated RT, other studies have shown promising results with high-dose, ablative techniques like SBRT. Our relatively favourable survival outcomes, achieved with a median BED_10_ of 59.5 Gy, suggest that effective local control and survival benefit are not exclusively limited to SBRT, although our results are not directly compared to patients receiving salvage SBRT. Given the relatively favourable outcomes of SBRT as well as the much shorter treatment time, we currently use ultrahypofractionated therapy in this scenario. Nonetheless, strategic conventional RT remains a valuable and effective option, especially when SBRT is unavailable or contraindicated.

It is important to recognize the various limitations of this study. There are inherent biases introduced by the retrospective nature of the research, especially in the patient selection and treatment administration. The population of patients described in this work has a relatively small burden of disease, which is localized, representing a small proportion of patients with recurrent pancreatic cancer at large. With respect to treatment benefit, it is difficult to isolate the impact of salvage RT because of the unequal distribution of post-recurrence chemotherapy administration between the two cohorts (75% in the salvage RT group versus 31.25% in the non-salvage RT group). Furthermore, the dominant chemotherapies given also differ significantly, adding further challenges to drawing sound conclusions. In the clinic, patient selection for radiotherapy in this setting remains challenging. Given the high propensity of this cancer for metastatic spread, decisions around patient selection must be made carefully in a multidisciplinary setting, with consideration to available systemic therapies, applicable trials, and all available modalities. In our practice, RT is most commonly recommended for individuals with locoregional recurrence who decline or are unsuitable for systemic therapies. This study also reflects the real-world lack of standardization in treating LLR. There are currently no consensus guidelines for target volume delineation, including the elective treatment of nodal basins, or optimal dose and fractionation schedules. However, it is worthwhile pointing out that pancreas SBRT contouring guidelines exist for the treatment of intact pancreatic cancers [14], and available reports also support coverage of nodal basins due to concerns over marginal recurrence [4]. Future prospective studies are needed to establish such standards to optimize outcomes and reduce variability in practice. Furthermore, the limited sample size restricts the findings’ generalizability, especially when considering an uncommon clinical circumstance. To validate these results and investigate the best way to combine salvage RT with other therapeutic modalities, future investigations should concentrate on including bigger patient cohorts. Examining the molecular and genetic characteristics of patients who benefit from salvage radiation therapy compared to those who do not may shed light on individualized treatment plans. Furthermore, research into the function of innovative systemic treatments in conjunction with salvage radiotherapy may contribute to a decrease in the frequency of distant metastases and enhance patient outcomes.

## 5. Conclusions

Radiotherapy is a potentially useful modality in the treatment of pancreatic cancer with isolated locoregional recurrence. Given the overall poor outcomes of such patients and risk for toxicity, treatment decisions should be made in a multidisciplinary setting with experienced providers. Careful consideration of patient factors and available therapies is key.

## Figures and Tables

**Table 1:** Baseline patient and tumour characteristics of two treatment groups (salvage RT and non-salvage RT).

	Salvage	Non-Salvage	Total	*p* Value
	(N = 16)	(N = 16)	(N = 32)	
Age at surgery (years)				
Mean (SD)	61.49 (9.43)	66.42 (11.55)	63.95 (10.67)	0.2
Median (Range)	60.6 (44.4–78.0)	67.0 (50.6–84.0)	61.4 (44.4–84.0)	0.32
Gender (Male = 0, Female = 1)				
0	08 (050.00%)	06 (037.50%)	14 (43.75%)	0.48
1	08 (050.00%)	10 (062.50%)	18 (56.25%)	
Type of surgery (specify; Whipple’s, etc.)				
Distal pancreatectomy	01 (6.25%)	03 (18.75%)	04 (12.50%)	0.6
Whipple	15 (93.75%)	13 (81.25%)	28 (87.50%)	
Location				
Body	01 (6.25%)	00 (0.00%)	01 (3.13%)	0.018
Head	15 (93.75%)	10 (62.50%)	25 (78.13%)	
Neck	00 (0.00%)	02 (12.50%)	02 (6.25%)	
Tail	00 (0.00%)	02 (12.50%)	02 (6.25%)	
Uncinate	00 (0.00%)	02 (12.50%)	02 (6.25%)	
Grade				
GI	04 (28.57%)	02 (14.29%)	06 (21.43%)	0.4
GII	10 (71.43%)	10 (71.43%)	20 (71.43%)	
GIII	00 (0.00%)	02 (14.29%)	02 (7.14%)	
Missing	02 (012.50%)	02 (012.50%)	04 (12.50%)	
Pathological TNM category				
T1	2 (12.50%)	1 (6.25%)	3 (9.38%)	0.19
T2	4 (25.00%)	6 (37.50%)	10 (31.25%)	
T3	10 (62.50%)	9 (56.25%)	19 (59.4%)	
N0	2 (12.50%)	2 (12.50%)	4 (12.50%)	
N1	12 (75.00%)	12 (75.00%)	24 (75.00%)	
N2	2 (12.50%)	2 (12.50%)	4 (12.50%)	
Margin status (Negative = 0; Positive = 1)				
0	16 (100.00%)	15 (93.75%)	31 (96.88%)	1
1	00 (0.00%)	01 (6.25%)	01 (3.13%)	
Number of nodes involved				
Mean (SD)	2.00 (2.00)	2.07 (1.58)	2.03 (1.78)	0.92
Median (Range)	1.5 (0.0–8.0)	2.0 (0.0–6.0)	2.0 (0.0–8.0)	0.6
Missing	16 (0)	15 (1)	31 (1)	
Number of nodes isolated				
Mean (SD)	21.75 (7.22)	17.40 (8.07)	19.65 (7.83)	0.12
Median (Range)	21.0 (13.0–36.0)	17.0 (2.0–31.0)	19.0 (2.0–36.0)	0.26
Missing	16 (0)	15 (1)	31 (1)	
Time from surgery to 1st Locoregional recurrence (years)				
Mean (SD)	1.94 (2.00)	1.28 (0.55)	1.61 (1.48)	0.22
Median (Range)	1.1 (0.5–7.0)	1.2 (0.4–2.3)	1.2 (0.4–7.0)	0.92
Resectability status at diagnosis (resectable/borderline/unresectable)				
Resectable	16 (100.00%)	15 (93.75%)	31 (96.88%)	1
Unresectable	00 (0.00%)	01 (6.25%)	01 (3.13%)	
Biochemical				
Ca 19-9 elevated	13 (81.25%)	14 (87.5%)		
Ca 19-9 Median (range)	84 (1-1245)	70 (11-3703)		0.29

**Table 2:** Pre-locoregional recurrence systemic therapy details of two treatment groups (salvage RT and non-salvage RT).

	Salvage	Non-Salvage	Total	*p* Value
	(N = 16)	(N = 16)	(N = 32)	
**Was chemotherapy given**				
Yes	16 (100.00%)	09 (56.25%)	25 (78.13%)	0.007
No	00 (0.00%)	07 (43.75%)	07 (21.88%)	
**If yes, adjuvant, neoadjuvant, or both**				
Adjuvant	13 (81.25%)	08 (88.89%)	21 (84.00%)	0.77
Both	02 (12.50%)	00 (0.00%)	02 (8.00%)	
Neoadjuvant	01 (6.25%)	01 (11.11%)	02 (8.00%)	
**Chemotherapy regimen (specify)**				
FOLFIRINOX	07 (43.75%)	01 (11.11%)	08 (32.00%)	0.24
Gemcitabine	06 (37.50%)	06 (66.67%)	12 (48.00%)	
Gemcitabine/Erlotinib	00 (0.00%)	01 (11.11%)	01 (4.00%)	
Gemcitabine/Capecitabine	02 (12.50%)	01 (11.11%)	03 (12.00%)	
Gemcitabine/Erlotinib	01 (6.25%)	00 (0.00%)	01 (4.00%)	
**Chemotherapy Duration, in months**				
Mean (SD)	4.7 (1.66)	1.69 (1.78)		<0.0001

**Table 3:** Pre-locoregional recurrence of two treatment groups (salvage RT and non-salvage RT).

	Salvage	Non-Salvage	Total	*p* Value
	(N = 16)	(N = 16)	(N = 32)	
**Size of largest disease focus**				
Mean (SD)	2.14 (0.65)	2.80 (0.96)	2.45 (0.86)	0.033
Median (Range)	2.1 (1.2–3.9)	2.6 (1.0–5.0)	2.5 (1.0–5.0)	0.023
Missing	16 (0)	14 (2)	30 (2)	
**Number of disease foci**				
Mean (SD)	1.50 (0.63)	1.21 (0.43)	1.37 (0.56)	0.16
Median (Range)	1.0 (1.0–3.0)	1.0 (1.0–2.0)	1.0 (1.0–3.0)	0.19
Missing	16 (0)	14 (2)	30 (2)	
**Regional (vascular, nodal)**				
Yes	14 (87.50%)	13 (81.25%)	27 (84.38%)	0.63
No	02 (12.50%)	3 (18.75%)	5 (15.63%)	
**Local (abutting surgical bed, remnant pancreas)**				
Yes	7 (43.75%)	5 (31.25%)	12 (37.50%)	0.46
No	9 (56.25%)	11 (68.75%)	20 (62.50%)	

**Table 4:** Dosimetric parameters of patients treated with salvage RT.

**Radiotherapy dose (BED_10_, in Gy)**	
Median (Range)	59.5 (39.0–61.7)
**Radiotherapy fractionation**	
Standard (1.8 Gy per fx)	12 (75%)
Hypofractionated (3.0 Gy per fx)	3 (18.8%)
SBRT (6.0 Gy per fx)	1 (6.3%)
**Concurrent chemotherapy**	
Oral Capecitabine	12 (75%)
None	4 (25%)
**Size of PTV (in cc)**	
Mean (SD)	363.07 (229.25)
Median (Range)	301.0 (240.8–485.2)
**Mean liver dose (in Gy)**	
Mean (SD)	7.72 (5.00)
Median (Range)	7.6 (5.1–10.4)
**D0.5cc to duodenum (BED_3_, in Gy)**	
Median (Range)	78.2 (6.7–82.9)
**D0.5cc to stomach (BED_3_, in Gy)**	
Median (Range)	55.8 (2.1–61.0)

**Table 5:** Key studies evaluating radiotherapy approaches for isolated locoregional recurrence of pancreatic cancer.

Author	Study Design	Sample Size	Median Radiation Dose/Fractionation	Local Control (LC)	Overall Survival (OS)	Distant Metastases-Free Survival (DMFS)
**Comito et al. (2017)** [7]	Retrospective analysis of SBRT	31	45 Gy in 6 fractions	91% (1 year), 82% (2 years)	Median: 18 months	Median PFS: 9 months
**Habermehl et al. (2013)** [11]	Retrospective analysis of CRT	41	Median 48.4 Gy (range: 39.6–54 Gy)	Median: 13.8 months	Median: 16.1 months; improved to 28.3 months with re-resection	Median PFS: 6.9 months
**Nakamura et al. (2014)** [1]	Retrospective analysis of RT	30	Median 50.4–54 Gy	67% (2 years)	Median: 15.9 months	Median PFS: 7.9 months
**Dee et al. (2024)** [4]	Retrospective analysis of ablative RT	65	BED_10_ ~98–100 Gy (e.g., 50 Gy/5 fractions)	72% (2 years, cumulative locoregional control)	Median: Not provided; 2-year OS: 57%	2-year DMFS: 22%
**Wilkowski et al. (2006)** [9]	Retrospective analysis of CRT	18	45 Gy in 25 fractions	28.9% local relapse rate	Median: 17.5 months (from CRT start); 27.2 months (from diagnosis)	Median PFS: 14.7 months
**Morris et al. (2021)** [12]	multi-institutional retrospective analysis	34	35 Gy (range 30–40 Gy) with a median BED of 59.5 Gy (α/β = 10)	6/34 patients (17.6%) experienced local failure as the first recurrence. No LC actuarial reported	OS 2y: 40%	Distant metastasis was the dominant failure pattern (65% of recurrences), but actuarial DMFS rates were not explicitly reported.
**Reddy et al. (2022)** [8]	Retrospective analysis	19	Median BED_10_: 54.8 Gy (range: 37.5–54.8 Gy)	LPFS: 1 and 2 years—63.2%, and 42.1%	17.1 months median OS; 71.3%, and 29.7% 1 and 2 years	15.6 months median DMFS 51.9%, and 31.1%—1 and 2 years
**Current Study**	Retrospective comparative analysis of RT	32	Median 50.4 Gy (range: 30–60 Gy, BED_10_ = 59.5 Gy)	62.5% maintained locoregional control post-RT	Median: 25.2 months (salvage RT) vs. 8.4 months (non-RT)	62.5% DM vs. 31.25% DM in non-RT group (*p* = 0.08)

## Data Availability

The data presented in this study are not available on request from the corresponding author due to ethics board considerations.
